# Aromatic Amines Exert Contrasting Effects on the Anticoagulant Effect of Acetaldehyde upon APTT

**DOI:** 10.1155/2014/735751

**Published:** 2014-12-08

**Authors:** La'Teese Hall, Sarah J. Murrey, Arthur S. Brecher

**Affiliations:** Department of Chemistry, Bowling Green State University, Bowling Green, OH 43403, USA

## Abstract

The pharmacological effects of amphetamine, procaine, procainamide, DOPA, isoproterenol, and atenolol upon activated partial thromboplastin time in the absence and presence of acetaldehyde have been investigated. In the absence of acetaldehyde, amphetamine and isoproterenol exhibit a procoagulant effect upon activated partial thromboplastin time, whereas atenolol and procaine display anticoagulant effects upon activated partial thromboplastin time. DOPA and procainamide do not alter activated partial thromboplastin time. Premixtures of procaine with acetaldehyde produce an additive anticoagulant effect on activated partial thromboplastin time, suggesting independent action of these compounds upon clotting factors. Premixtures of amphetamine with acetaldehyde, as well as atenolol with acetaldehyde, generate a detoxication of the anticoagulant effect of acetaldehyde upon activated partial thromboplastin time. A similar statistically significant decrease in activated partial thromboplastin time is seen when procainamide is premixed with acetaldehyde for 20 minutes at room temperature. Premixtures of DOPA and isoproterenol with acetaldehyde do not affect an alteration in activated partial thromboplastin time relative to acetaldehyde alone. Hence, a selective interaction of atenolol, procaine, and amphetamine with acetaldehyde to produce detoxication of the acetaldehyde is suggested, undoubtedly due to the presence of amino, hydroxyl, or amide groups in these drugs.

## 1. Introduction

The physiological and pharmacological effects of alcohol upon brain and CNS function have been the subject of many studies. Ethanol is readily metabolized in the body to CO_2_ and H_2_O by well-established enzymatic pathways (reviewed in [1]). Acetaldehyde (AcH), the primary metabolite in ethanol metabolism, is a highly reactive molecule which reacts readily with nucleophiles [2–4]. Among these compounds are amines, amides, imidazoles, thiols, and hydroxyls which are found in proteins, nucleic acids, select carbohydrates, and lipids. In an earlier communication, it was reported that highly reactive biogenic amine hormones, such as dopamine, epinephrine, serotonin, norepinephrine, and histamine, each of which contain primary or secondary amines, or hydroxyl groups, appear to readily react with AcH at room temperature (RT) and to “detoxify” the AcH, as evidenced by their capacity to reduce the anticoagulant effect of AcH on activated partial thromboplastin time (APTT) upon preincubation of the amines with AcH at room temperature (RT) [5]. It was further suggested that covalent interaction of the AcH with the biogenic amines would also “neutralize” their hormonal influences. As a consequence of these earlier investigations a new study of the effect of drugs with neurological impacts upon AcH, as followed by APTT, was initiated. In the study presented herein, the effects of amphetamine, procaine, procainamide, atenolol, and isoproterenol upon AcH are explored and compared with that of DOPA, the biological precursor to catecholamines and melanin. The ability of AcH-exposed drugs to prolong coagulation times, as a measure of pharmacological function, was investigated.

## 2. Materials and Methods

Procainamide, lot #54F-0048, procaine-HCl, lot #125K0697, isoproterenol, D-amphetamine, L-DOPA, lot #077K1844, and 5-atenolol, lot #044K3485, were purchased from Sigma-Aldrich Corporation, St. Louis, MD. APTT reagent, lot #2006-02-02/527313A, 0.025 $\underline{\underline{\text{M}}}$ CaC1_2_, lot #05-17-2004/5006872, Ci-Trol coagulation control, Level I, lots #2009-02-05/508114, 2006-01-19/538162/245, and 2010-06-07/548137/255, were obtained from Dade Behring, Marburg. Assess TM Level I plasma lot #N1106614 was obtained from Instrumentation Laboratory Company, Lexington, MA. Brockman I aluminum oxide, lot #0791DY, was purchased from Aldrich Chemical Company, Milwaukee, WI. Acetaldehyde (AcH), 99%, batch #00339MB, was secured from Sigma-Aldrich and passed through short columns of aluminum oxide in order to remove oxidation products. It was subsequently stored under N_2_ at −20°C until further use.

APTT assays were carried out as originally described elsewhere [6]. A fibrosystem fibrometer precision coagulation timer, model 5, Becton, Dickinson and Company, Cockeysville, MD, was employed to assay the APTTs.

## 3. Methods

### 3.1. Effect of Amphetamine Concentration on APTT

To 90 *μ*L of reconstituted plasma was added 10 *μ*L of ISB or 10 *μ*L of 10^−1^, 10^−2^, or 10^−3^ $\underline{\underline{\text{M}}}$ amphetamine. The solutions were mixed in fibrometer cups and stored for 20 min at RT. Subsequently, 100 *μ*L of APTT reagent at 37°C was added thereto and the mixtures were incubated at 37°C for an additional five min, after which time 100 *μ*L of 0.025 $\underline{\underline{\text{M}}}$ CaCl_2_ at 37°C was delivered in order to initiate the clotting reaction.

### 3.2. Effect of Amphetamine and AcH upon APTT

To 90 *μ*L of reconstituted plasma was added 20 *μ*L of ISB to serve as a control. To a second 90 *μ*L aliquot of plasma were added 10 *μ*L of ISB and 10 *μ*L of 0.1 $\underline{\underline{\text{M}}}$ amphetamine to give a final concentration of 4.5 × 10^−2^ $\underline{\underline{\text{M}}}$ amphetamine in plasma. To a third aliquot of plasma were added 10 *μ*L of 447 m$\underline{\underline{\text{M}}}$ AcH and 10 *μ*L ISB, to give an AcH concentration of 20.3 m$\underline{\underline{\text{M}}}$ in plasma. Lastly, to a fourth aliquot of 90 *μ*L of plasma was added 20 *μ*L of a mixture of equal volumes of amphetamine and 447 m$\underline{\underline{\text{M}}}$ AcH (which was stored for five min at RT). Each sample was mixed and stored for 20 min at RT, after which time 100 *μ*L of APTT reagent at 37°C was added thereto. The mixture was subsequently stored at 37°C for five min prior to initiation of clotting upon addition of 100 *μ*L of 0.025 m$\underline{\underline{\text{M}}}$ CaCl_2_ at 37°C.

### 3.3. Effect of Isoproterenol Concentration on APTT

To fibrometer cups containing 90 *μ*L of reconstituted Level I human plasma were added 10 *μ*L of ISB and 10 *μ*L of 10^−1^ $\underline{\underline{\text{M}}}$, 10^−2^ $\underline{\underline{\text{M}}}$, or 10^−3^ $\underline{\underline{\text{M}}}$ isoproterenol in ISB. The solutions were mixed, stoppered, and stored for 20 min at RT. Subsequently, 100 *μ*L APTT reagent was added thereto at 37°C and the mixture was further stored at 37°C for five min. Lastly, 0.025 $\underline{\underline{\text{M}}}$ CaCl_2_ (100 *μ*L) at 37°C was added to initiate clotting.

### 3.4. Effect of Isoproterenol and AcH upon APTT

To 90 *μ*L aliquots of plasma in fibrometer cups was added 20 *μ*L ISB or three alternative mixtures: (1) 10 *μ*L ISB + 10 *μ*L 10^−1^ $\underline{\underline{\text{M}}}$ isoproterenol; (2) 10 *μ*L ISB + 10 *μ*L 223.5 m$\underline{\underline{\text{M}}}$ AcH; and (3) 20 *μ*L of a 1 : 1 mixture of 10^−1^ $\underline{\underline{\text{M}}}$ isoproterenol and 223.5 m$\underline{\underline{\text{M}}}$ AcH. The solutions were mixed, stoppered, and stored at RT for 20 min, after which time 100 *μ*L of APTT reagent at 37°C was added thereto. After further storage at 37°C for five min, 100 *μ*L of 0.025 $\underline{\underline{\text{M}}}$ CaCl_2_ at 37°C was added in order to initiate clotting.

### 3.5. Effect of Atenolol and AcH upon APTT

To six 90 *μ*L aliquots of Level I plasma in fibrometer cups was added either 20 *μ*L ISB (1° control); 10 *μ*L ISB and 10 *μ*L of 0.075 $\underline{\underline{\text{M}}}$ atenolol (in 30% ethanol); 10 *μ*L ISB and 10 *μ*L of 223 m$\underline{\underline{\text{M}}}$ AcH; 10 *μ*L of 0.075 $\underline{\underline{\text{M}}}$ atenolol, followed five minutes later by 10 *μ*L of 223 m$\underline{\underline{\text{M}}}$ AcH; 10 *μ*L of 223 m$\underline{\underline{\text{M}}}$ AcH, followed five minutes later by 10 *μ*L of 0.075 $\underline{\underline{\text{M}}}$ atenolol; or 20 *μ*L of a 1 : 1 mixture of atenolol: AcH which had been stored at RT for five min. Upon standing at RT for five min, 100 *μ*L of APTT reagent was added thereto and the mixture was incubated at 37°C for an additional five min. Subsequently, clotting was initiated by addition of 100 *μ*L of 0.025 $\underline{\underline{\text{M}}}$ CaCl_2_ at 37°C. In separate experiments, the effect of atenolol-treated plasma was compared to control plasma containing approximately 1% ethanol.

### 3.6. Effect of Procainamide, Procaine, DOPA, and AcH upon APTT

To six fibrometer cups were added 90 *μ*L of plasma and each of the following: (1) 20 *μ*L ISB; (2) 10 *μ*L ISB and 10 *μ*L of 0.05 $\underline{\underline{\text{M}}}$ procainamide (to a final concentration of 4.5 m$\underline{\underline{\text{M}}}$ in plasma); (3) 10 *μ*L ISB and 10 *μ*L of 223.5 $\underline{\underline{\text{M}}}$ AcH (to a final concentration of 20.3 m$\underline{\underline{\text{M}}}$ in plasma); (4) ten *μ*L of procainamide, followed ten minutes later by 10 *μ*L of AcH; (5) ten *μ*L of AcH, followed ten minutes later by 10 *μ*L of procainamide; (6) 20 *μ*L aliquot of a 1 : 1 mixture of procainamide and AcH which had been previously stored and stoppered at RT for ten min. After standing at RT for ten min, 100 *μ*L of APTT reagent at 37°C was added thereto, and the cups were incubated at 37°C for five min. Subsequently, clotting was initiated upon addition of 100 *μ*L of 0.025 $\underline{\underline{M}}$ CaCl_2_. Four additional fibrometer cups contained a control stored at RT for 20 min, as well as plasma-procainamide, plasma-AcH, and plasma/procainamide-AcH premixtures stored for 20 min at RT. In an analogous manner, the effects of procaine and DOPA upon AcH were studied utilizing 9.1 m$\underline{\underline{\text{M}}}$ procaine in plasma and 0.9 m$\underline{\underline{\text{M}}}$ DOPA in plasma (due to solubility limitations).

### 3.7. Statistical Analyses

The data were analyzed by application of student's *t*-test. *P* values ≤ 0.05 were assumed to be statistically significant. In all groups of experiments, *n* = 4,5, 6,7, or 10, as indicated above in Methods section.

## 4. Results

The neurohormone, DOPA, and the neurotropic drugs, isoproterenol, atenolol, amphetamine, procaine, and procainamide each contain the benzene ring and various structural modifications. Each one presents a diverse picture relating to its effect upon APTT and its influence on the anticoagulant effect of AcH as a consequence of the presence/absence of functional groups which may interact with AcH. In essence, it was observed that amphetamine and isoproterenol exhibit a procoagulant effect upon APTT whereas atenolol and procaine display an anticoagulant effect upon APTT. Procainamide and DOPA have no statistical effect upon APTT under the conditions employed. AcH prolongs the APTT. Premixtures of amphetamine, atenolol, procaine, and procainamide with AcH exhibit a “detoxication” of the anticoagulant effect of AcH, that is, reduction in the anticoagulant activity of AcH. Successive additions of atenolol and AcH to plasma produce an additive anticoagulant effect, as does procaine. Successive additions of procainamide and AcH to plasma do not exhibit an additive anticoagulant effect with a 10-min exposure to plasma.

### 4.1. Effect of Atenolol and Acetaldehyde upon APTT

**Figure 1:**
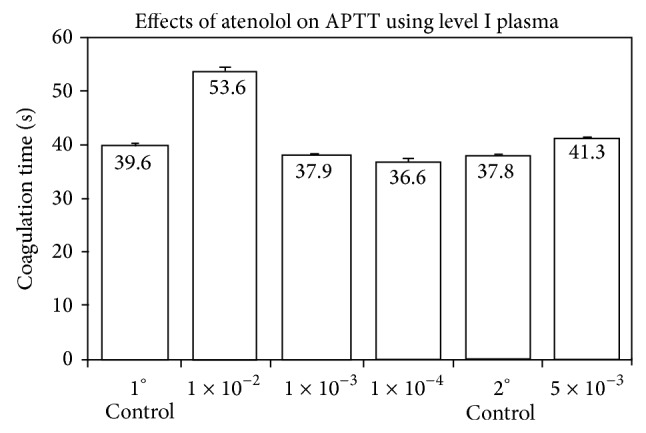
Effects of atenolol upon APTT using Level I plasma (*n* = 4; *P* = 0 for 1 × 10^−2^ $\underline{\underline{\text{M}}}$ and 5 × 10^−3^ $\underline{\underline{\text{M}}}$ atenolol) (*n* = 4; *P* = 0.07 for 1 × 10^−3^ $\underline{\underline{\text{M}}}$ atenolol and *P* = 0.01 for 1 × 10^−4^ $\underline{\underline{\text{M}}}$ atenolol).

**Figure 2:**
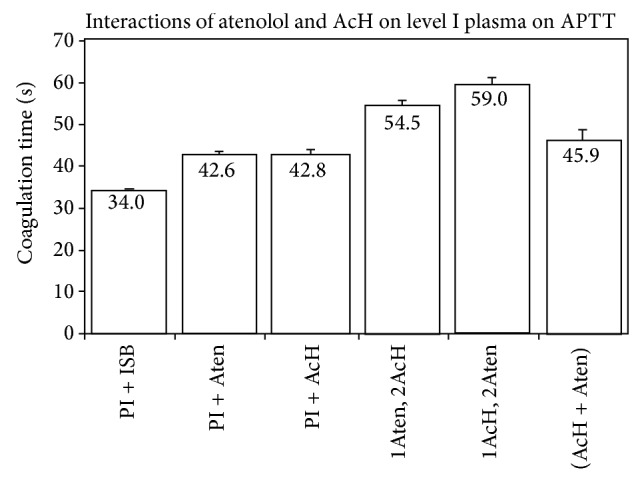
Interactions of atenolol and AcH on Level I plasma on APTT (*n* = 4; *P* = 0.002 for atenolol and for AcH; *P* = 0 for 1 Aten/2 AcH and for 1 AcH/2 Aten; *P* = 0.02 for the AcH and Aten premixture).

Atenolol additions to plasma to concentrations of 10^−2^ $\underline{\underline{\text{M}}}$ and 5 × 10^−3^ $\underline{\underline{\text{M}}}$ produced APTTs of 53.6 ± 0.8 sec (*n* = 4; *P* = 0) and 41.3 ± 0.1 sec (*n* = 4; *P* = 0) relative to their respective controls of 39.7 ± 0.6 and 37.8 ± 0.3 sec (*n* = 4) (Figure 1). At concentrations of 1 × 10^−3^ $\underline{\underline{\text{M}}}$ and 1 × 10^−4^ $\underline{\underline{\text{M}}}$, a slight procoagulant effect was observed with *P* = 0.07, approaching significance for the former concentration and a statistically significant *P* = 0.01 for the latter concentration. In studies involving both atenolol and acetaldehyde, it was noted that 7.5 × 10^−3^ $\underline{\underline{\text{M}}}$ atenolol in plasma and 20.3 m$\underline{\underline{\text{M}}}$ acetaldehyde in plasma each prolonged APTT, with values of 42.6 ± 0.7 (*P* = 0.002) and 42.8 ± 0.9 sec (*P* = 0.002), respectively, relative to a control of 34.0 ± 0.4 sec (*n* = 4) (Figure 2). Preincubation time of plasma with the drug was 5 min at RT. Hence, atenolol and acetaldehyde each exhibited anticoagulant effects. When atenolol was added initially to plasma for five min at RT and acetaldehyde was subsequently added for an additional five min prior to concluding the APTT, a value of 54.5 ± 0.8 sec (*P* = 0) was obtained. The atenolol and AcH effects on plasma were additive. When the order of addition of the reagents to plasma was reversed, an APTT of 59.0 ± 1.8 sec (*P* = 0) was noted. However, when atenolol and acetaldehyde were mixed and preincubated at RT for five min prior to addition to plasma for an additional five min at RT before concluding the APTT, an APTT of 45.9 ± 2.4 sec (*P* = 0) was observed. The drop in APTT from 54.5″ to 45.9″ suggested that atenolol partially inactivated the effect of acetaldehyde by exerting a detoxifying effect. The increase in APTT by the addition of acetaldehyde to plasma prior to addition of atenolol reflected the effect of increased exposure time of plasma to acetaldehyde.

### 4.2. Effect of Amphetamine and Acetaldehyde

**Figure 3:**
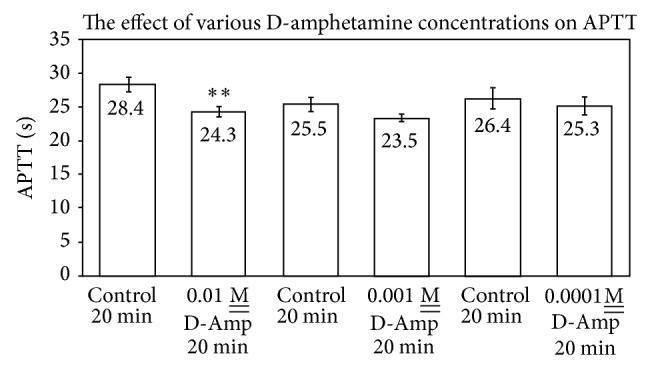
Effect of various D-amphetamine concentrations on APTT (*n* = 5; *P* ≤ 0.02 for 0.01 $\underline{\underline{\text{M}}}$ D-Amp) (*n* = 10; *P* ≤ 0.01 for the 20 min D-Amp-AcH mixture). ∗∗ indicate that *P* ≤ 0.05 relative to the control APTTs.

**Figure 4:**
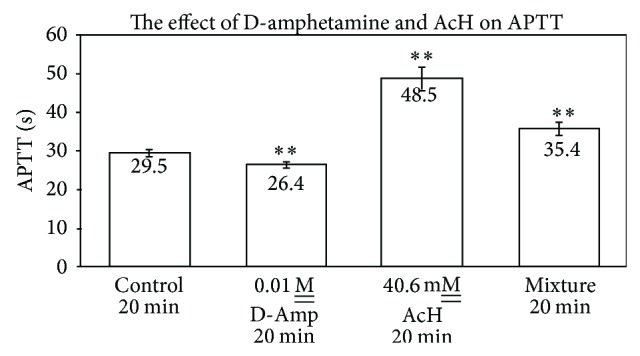
The effect of D-amphetamine and AcH on APTT (*n* = 10; *P* ≤ 0.01 for the D-Amp-AcH mixture).

Figure 3 indicates that 0.01 $\underline{\underline{\text{M}}}$ D-amphetamine exerted a statistically significant procoagulant effect upon APTT upon preincubation with plasma for 20 min at RT, with an APTT of 24.3 ± 0.8 sec (*P* ≤ 0.02) relative to a control of 28.4 ± 1.1 sec (*n* = 5). At 10^−3^ and 10^−4^ $\underline{\underline{\text{M}}}$ levels of amphetamine, there was no significant difference between control and experimental values. In an examination of the interactive effect of amphetamine and acetaldehyde upon the APTT reaction, it was observed that 0.01 $\underline{\underline{\text{M}}}$ amphetamine, upon exposure to plasma for 20 min at RT, exhibited an APTT of 26.4 ± 0.8 sec relative to a control plasma of 29.5 ± 0.9 sec, whereas 40.6 m$\underline{\underline{\text{M}}}$ acetaldehyde under the same conditions affected an APTT of 48.5 ± 2.2 sec (Figure 4). A premixture of acetaldehyde and amphetamine, standing at RT for 20 min prior to incubation with plasma for 20 min, resulted in an APTT of 35.4 ± 1.6 sec (*n* = 10, *P* ≤ 0.01) corresponding to a decrease of 13.1 sec in clotting time and a detoxication of the acetaldehyde by amphetamine.

### 4.3. Effect of Isoproterenol and Acetaldehyde upon APTT

**Figure 5:**
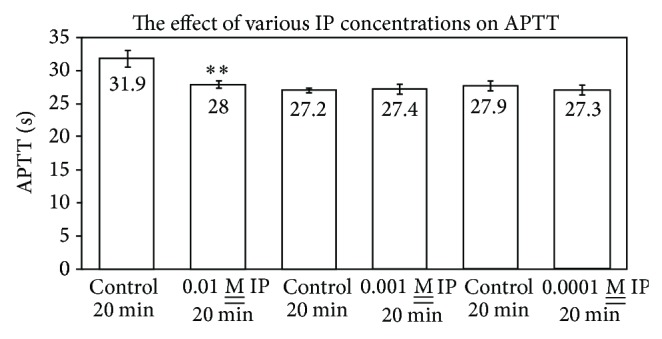
Effect of various isoproterenol concentrations upon APTT (*n* = 4; *P* = 0.03 for 0.01 $\underline{\underline{\text{M}}}$ IP).

**Figure 6:**
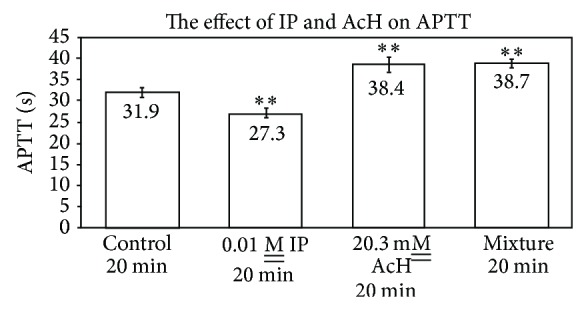
Effect of isoproterenol and acetaldehyde on APTT (*n* = 6; *P* = 0.01).

As indicated in Figure 5, 1 × 10^−2^ $\underline{\underline{\text{M}}}$ isoproterenol exerts a small but statistically significant procoagulant effect upon APTT since its clotting time is 28.0 ± 0.6 sec relative to the control of 31.9 ± 1.3 sec (*n* = 4, *P* = 0.03). At concentrations of isoproterenol at 10^−3^ and 10^−4^ $\underline{\underline{\text{M}}}$, no statistical difference is seen as compared to controls. The data in Figure 6 show that a premixture of isoproterenol and acetaldehyde exhibits essentially identical APTTs as acetaldehyde alone, with values of 38.7 ± 1.1 sec and 38.4 ± 1.8 sec, relative to the control of 31.9 ± 1.1 sec. In this series (*n* = 6, *P* = 0.01), the isoproterenol exhibited a procoagulant effect of 27.3 ± 1.1 sec. Hence, isoproterenol did not apparently react with acetaldehyde or affect its toxicity on APTT.

### 4.4. Effect of Procaine and Acetaldehyde upon APTT

**Figure 7:**
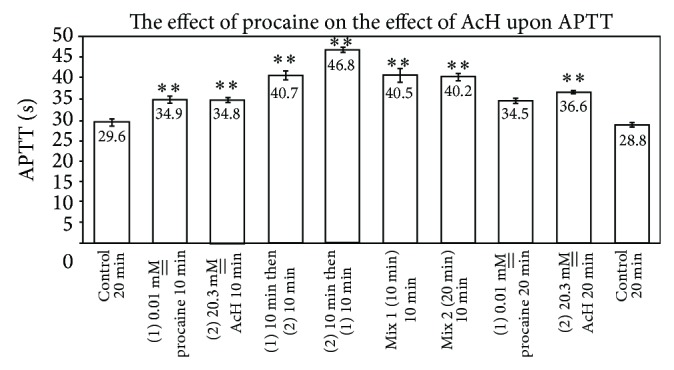
Effect of procaine and acetaldehyde upon APTT (*n* = 6; *P* = 0.001).

Figure 7 shows that 0.01 $\underline{\underline{\text{M}}}$ procaine and 20.3 m$\underline{\underline{\text{M}}}$ acetaldehyde each prolong APTT, relative to control APTT of 29.6 ± 0.8 sec, with APTTs of 34.9 ± 0.9 and 34.8 ± 0.5 sec, respectively (*P* = 0.001, resp.), when preincubation times of procaine or acetaldehyde are for 10 min at RT, indicating anticoagulant effects (*n* = 6). When procaine and acetaldehyde at the same concentrations are preincubated with plasma for 20 min at RT, the respective APTTs are 34.5 ± 0.6 sec and 36.6 ± 0.3 sec, relative to a control of 28.8 ± 0.5 sec (*P* = 0.0001, resp.). The increase in the anticoagulant effect of acetaldehyde with longer preincubation times with plasma has been previously reported [6]. Premixtures of procaine with acetaldehyde, upon storage for 10 or 20 min at RT before addition to plasma for an additional 10 min at RT before assay for APTT, resulted in APTTs of 40.5 ± 1.6 sec and 40.2 ± 0.9 sec, respectively (*P* = 0.0001, resp.). These essentially reflect an additive effect of each drug upon the components of the coagulation cascade. When procaine is added to plasma first for a ten-minute preincubation, followed by acetaldehyde second for an additional ten minutes, an APTT of 40.7 ± 1.0 sec is noted. When the order of addition to plasma is reversed, an APTT of 46.8 ± 0.6 sec is obtained, reflecting an increase in clotting time as a consequence of prolonged exposure of plasma to acetaldehyde.

### 4.5. Effect of Procainamide and Acetaldehyde upon APTT

**Figure 8:**
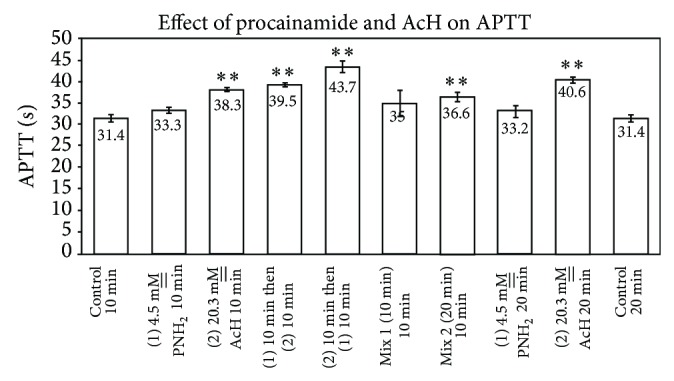
Effect of procainamide and acetaldehyde upon APTT (*n* = 6; *P* = 0.0001 for AcH; for 1 procainamide, 2 AcH; and for 1 AcH, 2 procainamide; *P* = 0.0035 for the 20 min premix of procainamide with AcH and is n.s. (0.3) for the 10 min premix).

Procainamide, 4.5 m$\underline{\underline{\text{M}}}$, and acetaldehyde, 20.3 m$\underline{\underline{\text{M}}}$, each prolonged APTT, relative to the control of 31.4 ± 0.9 sec, with APTTs of 33.3 ± 0.6 sec and 38.3 ± 0.4 sec, respectively, when preincubated with plasma at RT for ten min (*n* = 6, *P* = 0.0001 for acetaldehyde). Whereas the acetaldehyde-containing plasma gave a statistically significant difference from the control, the procainamide value was not statistically different (*P* = 0.1) (Figure 8). When incubations of procainamide and acetaldehyde with plasma were extended to 20 min at RT, APTTs of 33.2 ± 1.4 sec and 40.6 ± 0.7 sec, respectively, relative to a control of 31.4 ± 0.8 sec were obtained. Acetaldehyde, again, affected a greater prolongation of clotting time in 20 min as compared to 10 min. The procainamide-containing plasma did not statistically significantly alter APTT in 20 min as compared to its control plasma. Whereas 20.5 m$\underline{\underline{\text{M}}}$ acetaldehyde added to plasma produced APTTs of 38.3 ± 0.4 and 40.6 ± 0.7 sec (*P* = 0.0001 in both cases) in 10 and 20 min, respectively, 10- and 20-min premixtures of procainamide with acetaldehyde generated APTTs of 35 ± 3.2 and 36.6 ± 1.1 sec, respectively. The latter (20 min) values exhibited statistical differences between acetaldehyde alone and the acetaldehyde-procainamide premixture suggesting a detoxication of acetaldehyde by procainamide as a consequence of interaction. When procainamide was preincubated with plasma for ten min at RT after which acetaldehyde was subsequently added thereto, an APTT of 39.5 ± 0.4 sec was seen. When the order of addition of the compounds was reversed, an APTT of 43.7 ± 1.4 sec was noted. The increase in APTT was a reflection of the increased time of exposure of plasma to acetaldehyde.

### 4.6. Effect of DOPA and Acetaldehyde on APTT

**Figure 9:**
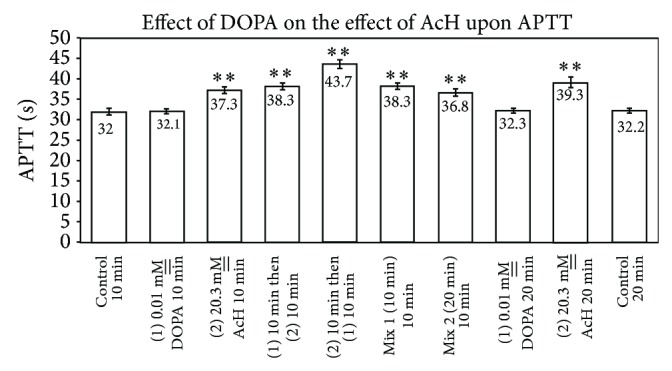
Effect of DOPA and acetaldehyde upon APTT (*n* = 7; *P* ≤ 0.05 relative to the controls).

Figure 9 shows that 0.01 $\underline{\underline{\text{M}}}$ DOPA does not significantly affect APTT after preincubation for 10 or 20 min (*n* = 7) (*P* = 0.92, n.s.). Whereas 20.3 m$\underline{\underline{\text{M}}}$ acetaldehyde significantly prolongs APTT upon preincubation with plasma for 10 min (*P* = 0.0001) as well as 20 min (*P* = 0.0002), premixtures of acetaldehyde with DOPA for 10 min or 20 min do not differ statistically from acetaldehyde alone in their APTT values. Preincubation of acetaldehyde alone with plasma gives a higher APTT after 20 min contact as compared to 10 min contact (39.3 sec relative to 37.3 sec), confirming all previous time course studies. Similarly, order of addition of reagents affected APTT since addition, first, of acetaldehyde to plasma with a 10-min contact time, followed by addition of DOPA thereto, gave an elevated APTT relative to the reverse order of addition (43.7 sec. relative to 37.3 sec), once again suggesting that longer contact times for interaction of acetaldehyde with plasma give greater prolongation of clotting times. Interestingly, however, it was observed that a 20-min premixture presented an APTT of 36.8 ± 1.0 sec whereas the APTT obtained when acetaldehyde was preincubated with plasma for 10 min prior to a second 10 min preincubation with added DOPA was 43.7 ± 1.0 sec, inferring that an interaction between acetaldehyde and DOPA has occurred in the 20-min preincubation time.

## 5. Discussion

The comparisons and contrasts between the structures as well as physiological and pharmacological effects of aromatic drugs such as amphetamine, isoproterenol, atenolol, procaine, and procainamide relative to the naturally occurring DOPA are marked. Furthermore, their response to interaction with AcH, the primary metabolite in alcohol metabolism, adds to the fascinating diversity in physiological and pharmacological responses. The comparison of these compounds with results obtained in earlier studies on such biogenic amine hormones as dopamine, epinephrine, norepinephrine, serotonin, and histamine also bears notice [5].

In contrast to the biogenic amines listed above (DA, E, NE, 5-HT, and H), all of the compounds in the current study, with the exception of the naturally occurring DOPA, namely, amphetamine, isoproterenol, atenolol, procaine, and procainamide, are drugs. Each is aromatic and each bears a broad resemblance to E, NE, and DA in that they contain the benzene ring. DOPA, procainamide, procaine, and amphetamine contain the primary amine group. Procaine, procainamide, and isoproterenol contain 2° N's, whereas atenolol, isoproterenol, and DOPA also contain hydroxyl groups (which should be capable of reacting with aldehydic functional groups). Procainamide, lastly, also contains a 1° amide group. Hence, many groups are susceptible to reactivity with AcH. Whereas each drug contains nucleophiles and rings, they differ amongst themselves in their ability to affect the APTT reaction. Accordingly, amphetamine and isoproterenol exhibit a procoagulant effect upon addition to plasma, whereas atenolol and procaine have an anticoagulant effect upon APTT. Procainamide and naturally occurring DOPA do not affect the APTT under the conditions employed. It should be noted that DA, the metabolic product of DOPA by decarboxylation, has a modest procoagulant effect upon APTT, as do EP and 5-H [5]. NE and histamine, however, do not statistically affect APTT [5].

Notwithstanding the similarities and diversities of the drugs studied herein with regard to nucleophilic properties and aromaticity, the difference in pharmacological effect upon APTT, relative to interactions of the drugs with AcH, is striking. Amphetamine, which exhibited a small, but statistically significant, procoagulant effect upon APTT, had a profound “detoxifying” effect upon APTT as a consequence of premixing the drug for 20 min at RT with AcH prior to addition to plasma (Figures 3 and 4). Essentially, the major anticoagulant effect of AcH upon APTT was markedly reduced by premixing with amphetamine. Atenolol, which exhibits an anticoagulant effect upon APTT, similarly statistically reduces the anticipated APTT when premixtures thereof with AcH are added to plasma. Individually, Aten and AcH each prolong APTT (Figure 2). Premixtures thereof are far less than additive in their APTT effect suggesting an interaction of the two, leading to a partial detoxication. When Aten is added to plasma prior to AcH, an APTT is recorded which is less than that when AcH is added first and Aten second. This is in agreement with earlier published results from this laboratory signifying that AcH affects a time-dependent increasing anticoagulant effect upon clotting time (PT). Although AcH reacts instantaneously with nucleophiles [2, 3], some reactions are reversible while others are irreversible [7, 8]. Presumably, this would explain increases and some slight ultimate decreases in coagulation times over extended periods of time, as tertiary structures of some proteins/enzymes are altered. Of further note is the fact that successive addition of Aten and AcH to plasma lends an additive effect upon APTT, suggesting that each of these components reacts at different sites on proteins, resulting in anticoagulant effects, when they do not react with one another. Whereas procainamide alone has no significant anticoagulant effect on APTT, successive additions of procainamide and AcH to plasma for 10 min at RT result in an anticoagulant effect corresponding to that of AcH alone and statistically significant (Figure 8). Upon premixing procainamide and AcH for 10 min prior to addition to plasma, there is no statistically significant reduction in the anticoagulant activity. However, when the premixture stands at RT for 20 min, a statistical drop in anticoagulant activity is observed, that is, a partial detoxication of the anticoagulant activity. This suggests that a slow reaction between AcH and procainamide, perhaps of a reversible nature, may be occurring. Of course, a reversible dissociation of AcH from the protein, with time, may also be occurring.

Procaine differs from procainamide in that the former exhibits an anticoagulant effect upon APTT, while successive additions of procaine and AcH to plasma produce an additive effect upon APTT, reflecting independent action of each compound upon protein components of the coagulation scheme (Figure 7). Interestingly, premixtures of procaine and AcH for 10 min, as well as 20 min, are reflected by an additive effect upon the APTT. This may be a reflection of the difference in reactivity of procaine, with an ester link, and procainamide, with an amide (2° amine) link.

Isoproterenol and DOPA, with their procoagulant and nonstatistical effects upon APTT, appear also to exert no statistical effect upon APTT when premixed with AcH (Figures 6 and 9). In the case of isoproterenol, AcH alone and AcH-IP mixtures show identical APTTs (Figure 6). In the DOPA experiments, successive additions behave as though DOPA was ineffective in influencing APTT in the presence of AcH. Similarly, 10-min and 20-min mixtures of AcH with DOPA behaved as if DOPA were absent (Figure 9). These data differ markedly from those with dopamine, which has a slight procoagulant effect and which decreases the anticoagulant effect of AcH when added successively to plasma as a premix with AcH [5]. The aromatic amines, epi, norepi, 5-HT, and histamine, each lower the anticoagulant effect of AcH upon plasma when preincubated with AcH at RT [5].

Clinically, the drugs exhibit powerful effects. Amphetamines, as well as methamphetamine (MDMA) and cocaine, are linked with increased blood pressure, cardiac arrhythmias, stroke, TIAs, infarctions, and hemorrhages [9, 10]. Amphetamines promote neurotransmission by blocking presynaptic uptake of catecholamines, thereby elevating their presence at the synapses, with the consequential saturation of postsynaptic receptors [10]. MDMA inhibits mitochondrial ALDH2 and cytosolic ALDH1 [11]. Long term serotonergic neurotoxicity of MDMA in rats is extended by ethanol as a result of the increased presence of acetaldehyde which inhibits ALDH1 and ALDH2 [12].

Isoproterenol, which is a *β*_1_-*β*_2_ adrenergic agonist, causes cardiac hypertrophy and myocardial infarction in mice and rats [13–20]. Galindo et al. [13] observed the modification of 865 genes by isoproterenol. Notably, however, caffeic acid lowers the extent of membrane damage by isoproterenol in male albino Wistar rats. Sesamol, neferine, and vitamin A protect rodents from damages by isoproterenol. Rats on an ethanol diet exhibit higher survival rates as a consequence of isoproterenol-induced MI. The alcoholic rats exhibited higher levels of ADH and ALDH [20].

The *β*-adrenergic blocker, atenolol, has antihypertensive, antianginal, and antiarrhythmic properties [21–27]. The antiarrhythmic effect is seen on administration of atenolol to rats with epinephrine-induced arrhythmia [26]. The combined application of ethanol and atenolol generates a reduced arrhythmic response in comparison to individual administration. Atenolol has been applicable for the treatment of ethanol-withdrawal syndrome [28, 29]. It also lowers the rate of reinfarction after an initial MI [23] thereby lowering mortality rates.

DOPA and dopamine are integrally related since DOPA is a metabolic product of tyrosine and dopamine is a sequential metabolic product of DOPA. Both are catecholamines and are precursors to norepinephrine and epinephrine. Together with 5-HT, they comprise biogenic amine hormones which decrease the anticoagulant effect of AcH upon APTT [5]. The most likely scenario involved is the formation of Schiff bases as well as hemiacetals and acetals between the AcH and the amines and hydroxyl moieties of these hormones, thereby detoxifying the AcH [5]. Johnson [30] noted in animal studies that lower levels of 5-HT are seen with an increase in alcoholic drinking. This may be reflected in the interaction of AcH with 5-HT to form Schiff bases. Sato et al. [31] have reported that patients treated with antiparkinsonism drugs who were on DOPA and dopamine agonists had elevated PTs. Among the most interesting developments in recent years is the report by Sandler et al. that 1-methyl-6,7-dihydroxy-1,2,3,4-tetrahydroisoquinoline, also known as salsolinol, was seen in the urine of parkinsonism patients under treatment with L-DOPA [32]. Further studies with rats and humans have led to the observation of salsolinol in rat adrenals and in the urine of alcoholics [33]. Salsolinol was reported to be a condensation product of dopamine and AcH [34]. N-Methyl salsolinol, a metabolic product of salsolinol, induced apoptosis in neurons [35] as a neurotoxin [36]. Hence, DOPA and dopamine engage both Parkinson's disease and AcH, although DOPA is not an agent utilizable for alcoholism [37].

Unlike the afore discussed metabolites and drugs, procaine and procainamide have not been explored for their interactions with alcoholism or acetaldehyde to any major extent. However, some effects of paraldehyde have been briefly studied in earlier years [38–40]. Interactions of biogenic amine hormones with AcH, as well as metabolic studies on their catabolic products, remain an ongoing field of interest.
